# Vitamin K_2_ and Cardiovascular Calcification: Mechanistic Foundations, Conflicting Clinical Evidence, and Unresolved Questions

**DOI:** 10.3390/ijms27219645

**Published:** 2026-10-28

**Authors:** Chinmayi Kothari, Rahul Chowdhary, Sada Babazade, Alexander Egoavil, Wajeh Abuirmaileh, Samir R. Kapadia, Sunil J. Wimalawansa

**Affiliations:** 1 Department of Cardiovascular Medicine, Heart, Vascular and Thoracic Institute, Cleveland Clinic, Cleveland, OH 44195, USA; 2 Department of Internal Medicine, Cleveland Clinic, Cleveland, OH 44195, USA; 3 Department of Medicine, Cardiometabolic & Endocrine Institute, North Brunswick, NJ 08902, USA

**Keywords:** matrix Gla protein (MGP), coronary artery calcium, valve calcification, bioprosthetic, valve degeneration

## Abstract

Cardiovascular calcification is an active, complex process involving osteogenic transformation, inflammation, extracellular vesicle release, and impaired mineral buffering. These changes result from mineral imbalance and reduced levels of calcification inhibitors. Vitamin K supports the γ-carboxylation of matrix Gla protein (MGP) and other mineralization inhibitors, though some calcification defenses do not depend on vitamin K. Vitamin K deficiency and warfarin therapy increase circulating dephosphorylated-uncarboxylated MGP (dp-ucMGP), which is linked to greater vascular calcification. Preclinical studies demonstrate that disruption of the vitamin K-MGP axis promotes calcification. Supplementation with vitamin K_1_ or K_2_ can reduce, prevent, or partially reverse mineralization in certain animal models. In contrast, evidence from human studies is less consistent. Observational studies have identified an association between menaquinone (vitamin K_2_) intake and cardiovascular outcomes, but causality has not been established. Most randomized trials have shown neutral results for aortic-valve and chronic kidney disease-associated calcification, while the VitaK-CAC and DANCODE studies reported slower progression of coronary artery calcium in selected populations with moderate or severe baseline coronary calcification. These discrepancies may be due to differences in calcification phenotypes, disease stage, vitamin K forms, dosing regimens, vitamin D status, renal function, and response to vitamin K antagonists such as warfarin. Coronary intimal, valvular, and arterial medial calcification should be regarded as phenotypically distinct entities. This review evaluates the mechanistic, preclinical, and clinical evidence linking vitamin K_2_ to cardiovascular calcification and highlights population groups that warrant further clinical investigation. Large, adequately powered, and phenotype-specific clinical trials are needed to determine whether appropriately dosed vitamin K_2_ is a safe and cost-effective intervention for slowing valvular and vascular calcification.

## 1. Introduction

Cardiovascular calcification refers to the deposition of calcium phosphate in arterial walls and cardiac valves. It has shifted from being an incidental imaging finding to an important prognostic marker in cardiovascular disease (CVD) [1,2]. Ectopic mineralization, once seen as a passive result of aging, is now recognized as an actively regulated, cell-mediated process involving several pathways, including the endogenous vitamin K recycling pathway described in Section 3 [3]. Soft tissue calcification develops over decades and serves as both a prognostic marker for atherosclerotic and degenerative disease and a potential therapeutic target [1,2].

Cardiovascular calcification includes several biologically distinct phenotypes. Phenotypes particularly relevant to the vitamin K-Matrix Gla Protein (MGP) axis are coronary intimal calcification, native valvular calcification in the aortic valve, chronic kidney disease-associated medial arterial calcification, and bioprosthetic valve mineralization [1,3,4]. Coronary artery calcification (CAC) refers to calcification within atherosclerotic plaques of the epicardial coronary arteries [2]. CAC can be quantified using non-contrast, electrocardiographically gated computed tomography imaging with the Agatston score [2]. It also independently predicts major adverse cardiac events more strongly than other non-invasive techniques used in contemporary risk calculators [2,5]. Calcific aortic valve disease involves fibrocalcific remodeling of the valves, spanning a continuum from aortic sclerosis to aortic stenosis. Although the underlying pathogenesis, including chronic inflammation, lipid deposition, and cellular trans-differentiation, is similar [1,6,7], calcific aortic valve disease differs from CAC in hemodynamic environment, cellular mediators, and clinical trajectories [1]. This distinction has important therapeutic implications.

### 1.1. Practical Implications in the Communities

In a registry of more than 25,000 asymptomatic patients, in those with a risk-adjusted CAC score ≥ 1000, 10-year survival declined from 99.4% to 87.8%, after adjustment for age and other traditional cardiovascular risk factors [8]. A score of zero and the extent of CAC predict mortality for up to 15 years in asymptomatic individuals [5]. The valvular mineralization burden rises with increasing age. In the contemporary PREVUE-VALVE study of adults aged 65–85 years, the weighted prevalence of aortic stenosis was 6.1%, including 3.1% with at least moderate disease [9]. Aortic valve replacement is the established treatment for severe symptomatic aortic stenosis [10].

### 1.2. Broader Role of Vitamin K_2_

Vitamin K_2_ (menaquinone) is a mechanistically relevant regulator of calcification. Menaquinone activates MGP by serving as a co-factor for its γ-carboxylation. Through this reaction, it activates a potent inhibitor of connective tissue matrix calcification (MGP) [3], as illustrated in animal studies. *MGP* gene-knockout mice die within two months as a result of arterial calcification leading to blood vessel rupture [11]. The use of vitamin K antagonists like warfarin results in an elevated proportion of uncarboxylated MGP residues, increasing the risk of valvular and vascular calcification [12,13].

The above-mentioned mechanistic hypothesis is partly supported by the VitaK-CAC randomized trial, in which two years of menaquinone-7 (360 µg/day) resulted in significantly (*p* = 0.02) less CAC progression and similarly less progression in calcium mass versus placebo [14]. Two analyses of the AVADEC trial, which administered MK-7 at 720 μg/day plus vitamin D_3_ at 25 μg/day for two years, found no reduction in established aortic valve calcification or overall coronary artery calcium progression [15]. However, less coronary calcium progression was observed in a subgroup with baseline CAC ≥ 400 Agatston units [16]. These findings suggest that treatment response may differ according to calcification phenotype and baseline burden.

This review examines whether biochemical activation of the vitamin K–MGP axis translates into meaningful modification of cardiovascular calcification. We integrate mechanistic, preclinical, observational, and randomized evidence while distinguishing coronary intimal calcification, native aortic valve calcification, CKD-associated medial calcification [17] and bioprosthetic valve degeneration. Attention is given to the discordance between biochemical target engagement, imaging outcomes, and clinical relevance. This narrative review did not use a prespecified systematic search strategy and does not report a new meta-analysis. Published meta-analyses are discussed as secondary evidence. Additional targeted literature searches conducted during manuscript revision are described in Appendix A. 

## 2. Clinical Significance of Cardiovascular Calcification

### 2.1. Valvular Calcification

Calcific aortic valve disease is a slowly progressive, active process rather than a passive degenerative process, as previously thought [18,19]. The spectrum of fibrocalcific changes ranges from aortic sclerosis to aortic stenosis. Aortic sclerosis involves mild leaflet thickening with focal mineralization and preserved hemodynamics. Aortic stenosis includes advanced calcification and impaired leaflet motion, causing left ventricular outflow tract obstruction [1,6].

The extent of calcific aortic valve disease observed on non-contrast CT predicts the long-term risk of progression to severe disease [20]. As calcification burden increases, transvalvular pressure gradients rise, and chronic pressure overload leads to left ventricular hypertrophy, which is associated with an elevated risk of heart failure, cardiac arrhythmia, and sudden cardiac death [1,6]. Due to the poor prognosis of severe, symptomatic aortic stenosis without valve replacement, this condition is now the most common indication for percutaneous or surgical valve replacement. Current management is guided by the 2025 ESC/EACTS guideline [10].

Current management strategies primarily address mechanical relief of obstruction and do not directly target the underlying pathogenetic mechanisms responsible for calcification. Chronic inflammation and progressive calcification form a self-perpetuating cycle. No medical therapy has been shown to halt or reverse established soft tissue mineralization [10,21]. Consequently, the potential therapeutic window for menaquinone likely occurs during the early stages of aortic sclerosis, when the calcium burden is mild and before the development of fixed obstruction and irreversible left ventricular remodeling [1,15].

### 2.2. Coronary Artery Calcification

Coronary artery calcification is a component of atherosclerotic plaque and is most commonly quantified on computed tomography using the Agatston score [2,5]. As mentioned above, CAC burden is a strong marker of future atherosclerotic cardiovascular disease and all-cause mortality. It can refine risk estimates and add value beyond traditional risk factors [2,5,8]. A score of zero identifies a low near-term event rate in many asymptomatic adults, whereas increasing scores indicate progressively greater plaque burden and risk. CAC score should nevertheless be interpreted as a measure of total calcified plaque burden rather than a direct measure of the degree of luminal stenosis.

The biology and clinical meaning of coronary calcification depend on the morphology and stage of calcification. Microcalcification within an inflamed fibrous cap may contribute to plaque vulnerability, whereas larger, confluent deposits may accompany plaque maturation and stabilization [22]. Although statin therapy may increase plaque calcification density, it significantly reduces lipid-rich plaque and cardiovascular events [23]. Therefore, a rising Agatston score during effective statin therapy should not be considered treatment failure [23]. This complicates the use of serial CAC progression as a surrogate outcome in vitamin K trials, particularly when most participants receive statins.

CAC also differs from predominantly medial calcification, as seen in advanced CKD. Intimal CAC develops within atherosclerotic plaque, whereas medial calcification involves the arterial media and is more closely associated with arterial stiffness and disturbances in mineral metabolism [22,24] associated with CKD mineral and bone disorder (CKD-MBD) [17]. Trials should therefore specify the vascular bed, calcification compartment, baseline burden, renal function, and background preventive therapy.

### 2.3. Shared Pathobiology

Calcification is an active process mediated by vascular smooth muscle cells and valvular interstitial cells, rather than a passive precipitation of excess mineral. These cells undergo an osteogenic phenotype switch, resulting in downregulation of contractile proteins and increased hydroxyapatite deposition [1,22,25]. The master osteogenic transcription factor RUNX2 (Cbfa1) is central to this transition; it represses smooth muscle differentiation and promotes mineralization. In contrast, smooth muscle-specific *RUNX2* deletion significantly inhibits vascular calcification in vivo [26,27].

Several upstream triggers for this phenotype switch are common to coronary arteries and valves. Oxidative stress (hydrogen peroxide and oxidized low-density lipoprotein) and accumulated deoxyribonucleic acid damage associated with aging activate RUNX2 through protein kinase B signaling and promote the contractile-to-osteogenic switch [22,27]. Recent experimental work also implicates ferroptosis, related to repression of the solute carrier family 7 member 11–glutathione–glutathione peroxidase 4 axis, in vascular smooth muscle cell calcification [28]. Pro-inflammatory cytokines such as tumor necrosis factor alpha, interleukin 1 beta, and interleukin 6, together with the receptor activator of nuclear factor kappa B ligand/osteoprotegerin axis and NF-κB signaling, activate osteogenic gene expression [1,22]. Contemporary studies have also described toll-like receptor 3 signaling as a contributor to valvular calcification [29]. Metabolic bone-mineral disorders such as CKD-MBD with increased calcium and phosphate promote ectopic mineralization [24]. Foremost among the inhibitors of this procalcific milieu is vitamin K-dependent MGP, whose inactivation promotes unopposed mineralization (Table 1).

Extracellular vesicles (EVs) connect cell phenotype to mineral nucleation. In VSMC experiments, sortilin-dependent trafficking of calcification-associated cargo, including tissue-nonspecific alkaline phosphatase (TNAP), promotes mineralizing EV activity [30]. Valvular sortilin signaling also links fibrosis and cellular heterogeneity to calcification [7]. EV behavior depends on cargo and context; not all vesicles are procalcific. This route is distinct from simply depositing calcium after loss of MGP.

Circulating mineral buffering provides another layer of control. Fetuin-A-containing calciprotein particles (CPPs) initially sequester calcium and phosphate; maturation toward more crystalline secondary particles can increase calcification stress. CPPs are mineral–protein complexes, not membrane-bound EVs. The T50 assay measures ex vivo particle-maturation time and serum calcification propensity, not tissue calcium burden or a validated therapeutic target [31]. Human CKD studies associate CPP burden with vascular remodeling, while experiments implicate GRP-dependent differences in particle and vesicle calcifying activity [32,33]. Particle abundance, composition, and maturation therefore should not be treated as interchangeable measurements.

Inorganic pyrophosphate (PPi) inhibits hydroxyapatite growth. Ectonucleotide pyrophosphatase/phosphodiesterase 1 (ENPP1) generates extracellular PPi, whereas TNAP hydrolyzes it, reducing this inhibitory brake. VSMC TNAP overexpression and ENPP1-replacement experiments support the importance of this axis in animal mineralization models [34,35]. These studies do not test oral vitamin K efficacy; they illustrate a parallel defense that MGP carboxylation alone need not restore.

Single-cell and spatial analyses of human calcified valves identify heterogeneous valvular interstitial cell and macrophage states and sex-associated differences in cellular programs [36]. These findings challenge the concept of calcific aortic valve disease as a uniform osteogenic lesion but do not establish sex-specific responses to vitamin K supplementation. Lipoprotein(a) is a separate, genetically supported valve-disease pathway [37]. The valve-specific pelacarsen study Lp(a)FRONTIERS CAVS (NCT05646381) is a phase 2 trial, distinct from phase 3 atherosclerotic cardiovascular disease (ASCVD) outcome programs; its registry is not a completed efficacy result [38]. These findings place vitamin K within a multifactorial disease framework.

**Table 1:** Comparison of coronary intimal, aortic valvular, medial arterial calcification and bioprosthetic valve degeneration.

Feature	Coronary Plaque Calcification	Native Aortic Valve Calcification	CKD-Associated Medial Arterial Calcification	Calcific Bioprosthetic Structural Valve Degeneration
Anatomic compartment	Arterial intimal plaque [22]	Valve leaflet matrix, mainly fibrosa [1]	Arterial media along elastic lamellae [22,39]	Fixed xenograft leaflets [4]
Principal cell types	VSMCs, endothelial cells, and osteogenic progenitor cells [22]	Valvular interstitial/endothelial cells and inflammatory cells [1]	VSMCs and circulating mineral complexes [22]	Devitalized tissue with infiltrating host cells [4,40]
Mechanical environment	Shear, pulsatile pressure, and plaque stress [22]	Repetitive flexion, tissue stress, and oscillatory shear [1]	Circumferential stretch and increasing stiffness [22]	Flexion, turbulent flow and focal attachment stress [4]
Initiating stimuli	Lipids, endothelial dysfunction, inflammation and plaque necrosis [22]	Lipid deposition, inflammation, oxidative stress and mechanical injury [1]	Mineral imbalance, uremia, metabolic disease and vitamin K antagonism [41]	Tissue-processing injury, immune reactions and mechanical fatigue [4]
Major molecular pathways	BMP–RUNX2 and inflammatory signaling drive osteogenic VSMC transformation [27]	BMP–RUNX2 and lipid-inflammatory signaling drive osteogenic valvular-cell transformation [1]	Phosphate–RUNX2, BMP, Wnt and alkaline phosphatase signaling with loss of MGP [22]	Dystrophic mineralization promoted by tissue damage, inflammation and matrix degradation [4,40]
Mineralization morphology	Microcalcifications progressing to dense plaques [22]	Nodular deposits and fibrocalcific thickening; sometimes bone formation [1]	Linear, circumferential deposits in the arterial media [39]	Nodular or confluent deposits with fibrosis and leaflet damage [4]
Imaging methods	electrocardiographically gated CT calcium scoring; CT angiography and selected advanced imaging [2]	Echocardiography and CT; PET for active mineralization [1]	Radiography or CT; pulse-wave velocity for arterial stiffness [22]	Echocardiography and CT; selected fluoroscopy or transesophageal imaging [4,42]
Clinical consequences	Indicates atherosclerotic burden and cardiovascular risk [2,8]	Progressive aortic stenosis, cardiac overload and heart failure [1,6]	Arterial stiffness, hypertension and increased cardiovascular risk [22,43]	Prosthetic stenosis or regurgitation leading to valve failure [42,44]
Potential role of MGP	Locally inhibits mineralization; importance varies by plaque stage [3]	Likely protects against valvular mineralization [3,45]	Major inhibitor; vitamin K deficiency promotes calcification [11,13]	Present, but its functional importance remains uncertain [40,46]
Expected reversibility	Low after macroscopic calcium develops [14,23]	Essentially irreversible without valve replacement [6,15]	Possibly modifiable early, but human evidence is limited [47,48]	Irreversible once structural degeneration develops [4,42]

## 3. Biology of Vitamin K

The vitamin K family comprises three main forms with approximately 12 distinct compounds. Vitamin K_1_, phylloquinone, is found primarily in green leafy vegetables; vitamin K_2_, menaquinones, are present in fermented foods, animal products, and some tissues following conversion of dietary vitamin K; and vitamin K_3_, menadione, which is synthetic and toxic at high doses [49]. Menaquinones have ~10 subtypes: MK-4 through MK-13 (bacterial origin, except MK-4). Vitamin K_2_ is by far the most diverse branch, with MK-4 and MK-7 being the most studied clinically [49,50].

The central biological role of vitamin K is to serve as a cofactor for γ-glutamyl carboxylase, which converts selected glutamate residues to γ-carboxyglutamate in vitamin K–dependent proteins [49,50]. This post-translational modification enables these proteins to bind calcium and function effectively. In the liver, vitamin K_1_ supports the γ-carboxylation of coagulation factors II, VII, IX, and X, as well as proteins C, S, and Z, thereby maintaining balanced hemostasis.

Intestinal bacteria synthesize menaquinones; however, the extent of their absorption and their quantitative contribution to human vitamin K requirements remain unclear [49]. In extra-hepatic tissues, vitamin K facilitates the γ-carboxylation of matrix Gla protein, which inhibits ectopic vascular and valvular calcification, and osteocalcin, which regulates bone mineralization [3,49]. Vitamin K undergoes continuous recycling via the vitamin K epoxide cycle, primarily involving vitamin K epoxide reductase complex subunit 1 (VKORC1) [51]. This recycling process accounts for the mechanism by which warfarin, a VKOR inhibitor, reduces functional vitamin K availability and increases concentrations of undercarboxylated vitamin K–dependent proteins [49]. Therefore, vitamin K biology encompasses roles in coagulation, bone and vascular health, cellular signaling, and the regulation of pathological mineralization [49,51,52].

### 3.1. Vitamin K Forms

Vitamin K belongs to a family of fat-soluble molecules that share a common 2-methyl-1,4-naphthoquinone ring and differ in the lipophilic side chain attached at the 3-position [49,53]. This structural difference divides the naturally occurring forms of the family into two classes with distinct origins, kinetics, and tissue distribution. Vitamin K_1_ (phylloquinone) is synthesized by plants and is the dominant dietary form. Following intestinal absorption, it is directed to the liver, where it supports the γ-carboxylation of coagulation factors. Vitamin K_2_ (menaquinones) comprises a series of vitamers designated MK^−n^, where *n* is the number of isoprenoid units in the side chain. Menaquinones are primarily of bacterial origin and are found in fermented foods and animal products [53].

Although they belong to the same subgroup, the menaquinone subtypes differ in sources, side-chain structure, and tissue distribution. MK-4 is present in animal tissues such as egg yolk, meat, and liver, and arises from endogenous conversion of dietary phylloquinone and menadione via the enzyme UbiA prenyltransferase domain-containing protein 1 rather than from bacterial fermentation [49]. MK-7 and other long-chain menaquinones (MK-8 and MK-9) are produced by bacterial fermentation and are concentrated in fermented foods such as certain cheeses and curds [49].

**Figure 1:**
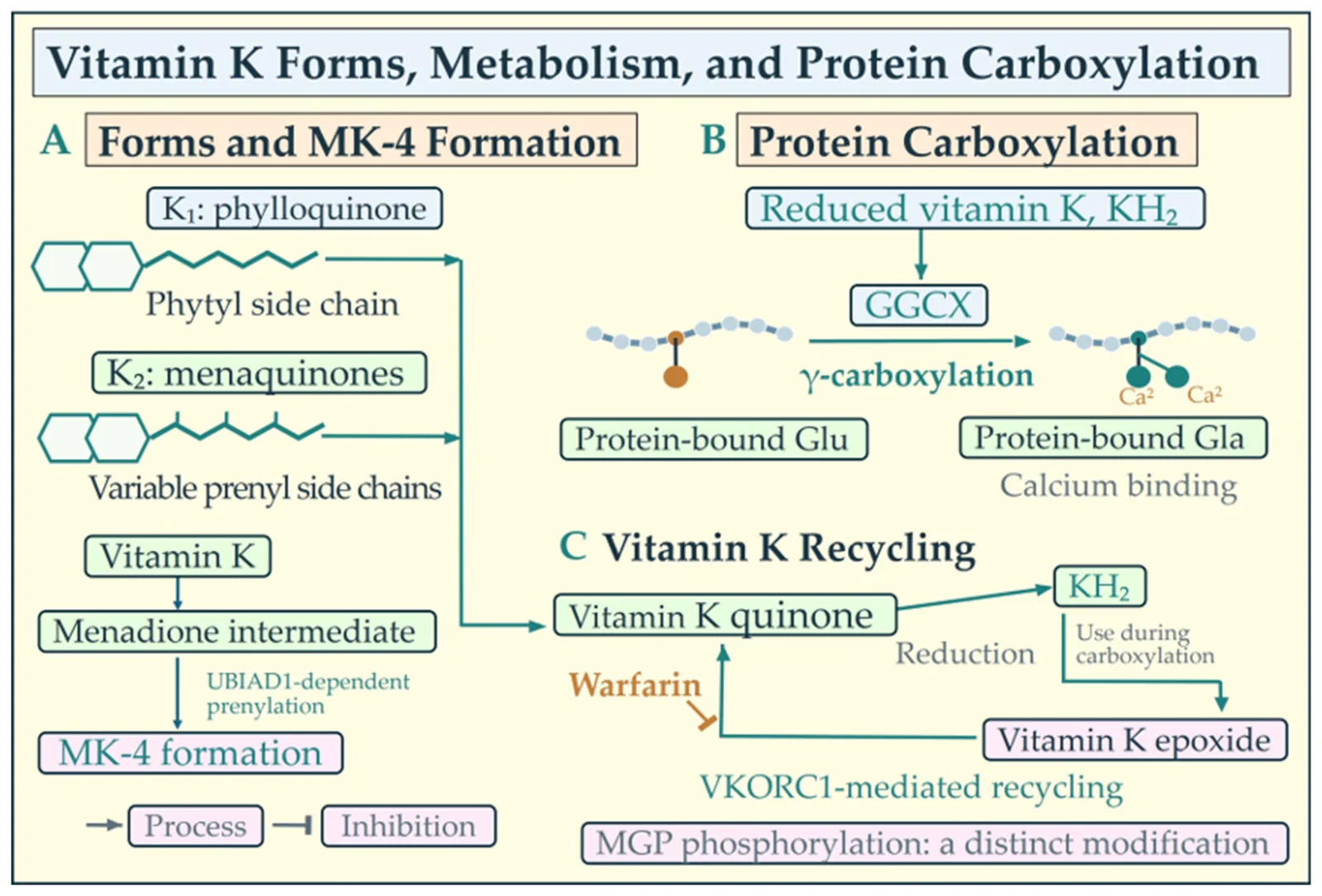
(**A**) Vitamin K forms, metabolism, and (**B**) protein carboxylation. K_1_ and (**C**) menaquinones share a quinone ring but differ in side chains. UbiA prenyltransferase domain-containing protein 1 participates in MK-4 formation through a menadione intermediate. Reduced vitamin K supports GGCX-mediated conversion of protein-bound glutamate to γ-carboxyglutamate (Gla); vitamin K is oxidized to its epoxide and recycled. Warfarin inhibits VKORC1-mediated recycling. MGP phosphorylation is a separate modification. The diagram is an original explanatory schematic, not a molecular structure or a quantitative pharmacokinetic model.

The traditional Japanese food natto, fermented with *Bacillus subtilis*, contains particularly high levels of MK-7. The side-chain length governs pharmacokinetics: increasing chain length raises lipophilicity, and MK-7 accordingly has a much longer plasma half-life of 72 h compared with roughly 3 h for MK-4 [52]. MK-7 is well absorbed, transported via lipoproteins, and redistributed to peripheral tissues, producing sustained circulating levels. In contrast, nutritional doses of MK-4 do not measurably raise the circulating MK-4 concentration [52]. Because of its longer half-life and superior extrahepatic bioavailability, MK-7 is the most relevant form for activating vitamin K-dependent proteins outside the liver, including those in the cardiovascular system. This pharmacokinetic profile explains the basis for the selection of MK-7 in most cardiovascular calcification trials [52,54]. Figure 1 illustrates different forms of vitamin K and their metabolic pathways.

### 3.2. Vitamin K-Dependent Proteins

Vitamin K is an essential co-factor for the post-translational γ-carboxylation of vitamin K–dependent proteins (VKDPs). In this reaction, the enzyme γ-glutamyl carboxylase (GGCX) converts specific glutamate residues to γ-carboxyglutamate (Gla) residues, which confer calcium-binding capacity [50,53]. GGCX utilizes KH2 (the reduced form of vitamin K) as a co-factor for this redox reaction, producing vitamin K epoxide. Vitamin K is subsequently recycled to KH2 by reactions involving vitamin K epoxide reductase complex subunit 1 (VKORC1) [49,51]. The utilization and regeneration of the active co-factor, KH2, constitutes the vitamin K cycle. Recycling permits vitamin K to participate repeatedly in γ-carboxylation reactions [49,51].

The VKDP family includes hepatic clotting factors (II, VII, IX, X) and anticoagulant proteins (C, S, Z) which support hemostasis. Extrahepatic VKDPs involved in mineral metabolism include MGP, a potent local inhibitor of soft tissue calcification that contains five Gla residues; osteocalcin, an osteoblast-derived protein involved in bone formation; and Gla-rich protein (GRP), a calcification inhibitor with an unusually high Gla content [3]. It is this extrahepatic arm that links vitamin K to cardiovascular calcification [51,53].

**Figure 2:**
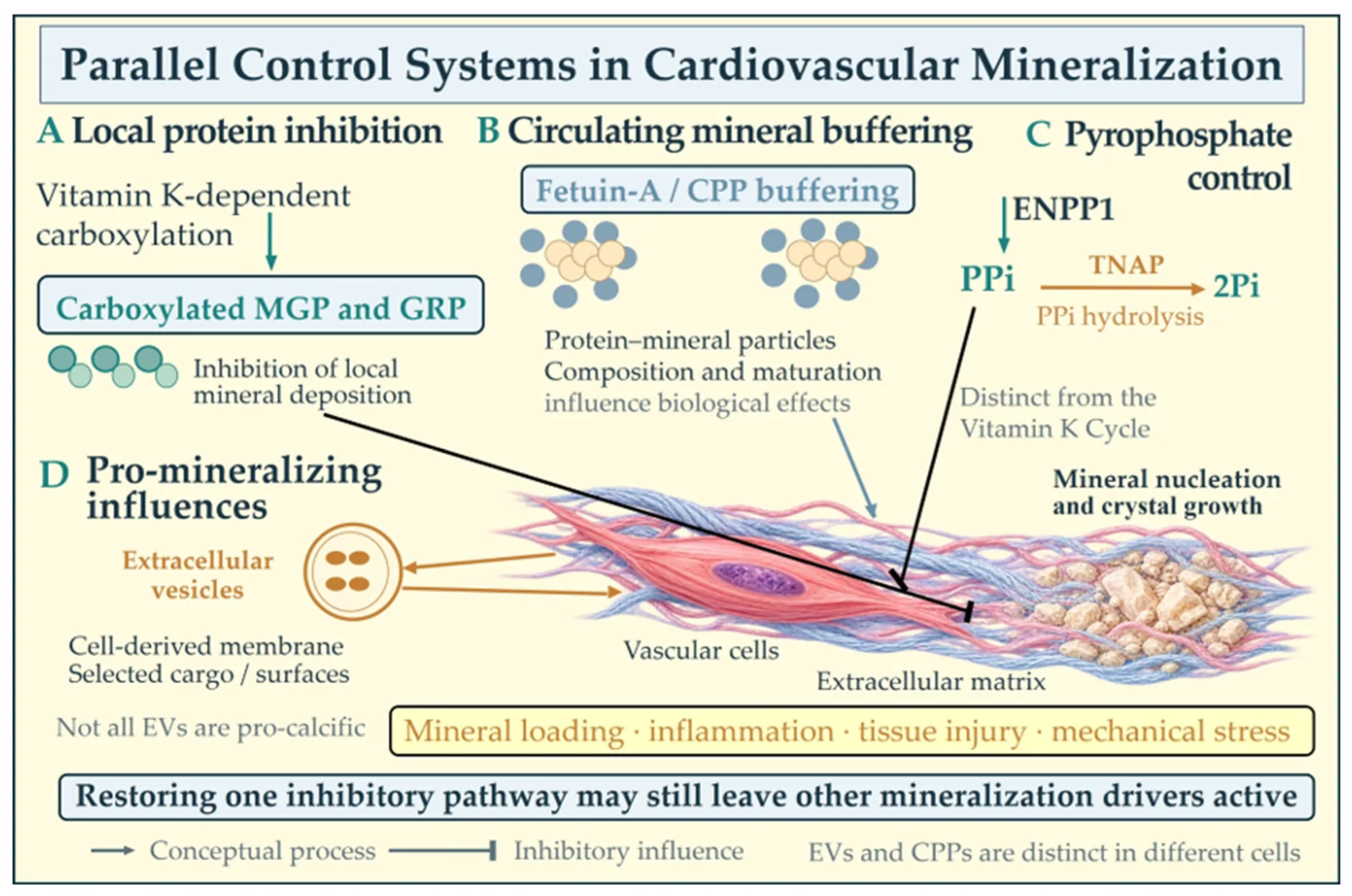
Parallel control systems in cardiovascular mineralization. (**A**) Local protein inhibition mediated by vitamin K–dependent carboxylation of matrix Gla protein (MGP) and Gla-rich protein (GRP), which inhibit mineral deposition. (**B**) Circulating mineral buffering by fetuin-A and calciprotein particles (CPPs), which regulate mineral handling. (**C**) Pyrophosphate regulation through ENPP1-mediated pyrophosphate (PPi) generation and tissue-nonspecific alkaline phosphatase (TNAP)–mediated PPi hydrolysis, influencing mineral nucleation. (**D**) Pro-mineralizing influences, including extracellular vesicles (EVs), mineral loading, inflammation, tissue injury, and mechanical stress. EVs and CPPs are distinct entities. Restoration of one inhibitory pathway may not normalize the entire network. Arrows denote conceptual processes, not proven treatment effects. Original explanatory schematic based on experimental and translational evidence [30,31,32,33,34,35].

GRP adds functional breadth to the vitamin K-dependent inhibitor system. Experimental work links carboxylated GRP to the mineral-binding environment of CPPs and EVs, including complexes containing fetuin-A and MGP [32,55]. This is evidence for an interacting inhibitory network, not proof that oral supplementation restores GRP function in human lesions or that a GRP-directed dose improves clinical outcomes [32]. Figure 2 illustrates the parallel control systems in cardiovascular mineralization.

### 3.3. Relevance to Cardiovascular Tissues

Vitamin K-dependent MGP is locally synthesized by vascular smooth muscle cells in the coronary arteries and by interstitial cells in valves [3,40]. It is upregulated in regions with a greater atherosclerotic burden and oxidative stress and actively inhibits hydroxyapatite deposition. Local levels of vitamin K influence GGCX-mediated activation of MGP and its downstream effects on calcification [3,13].

**Figure 3:**
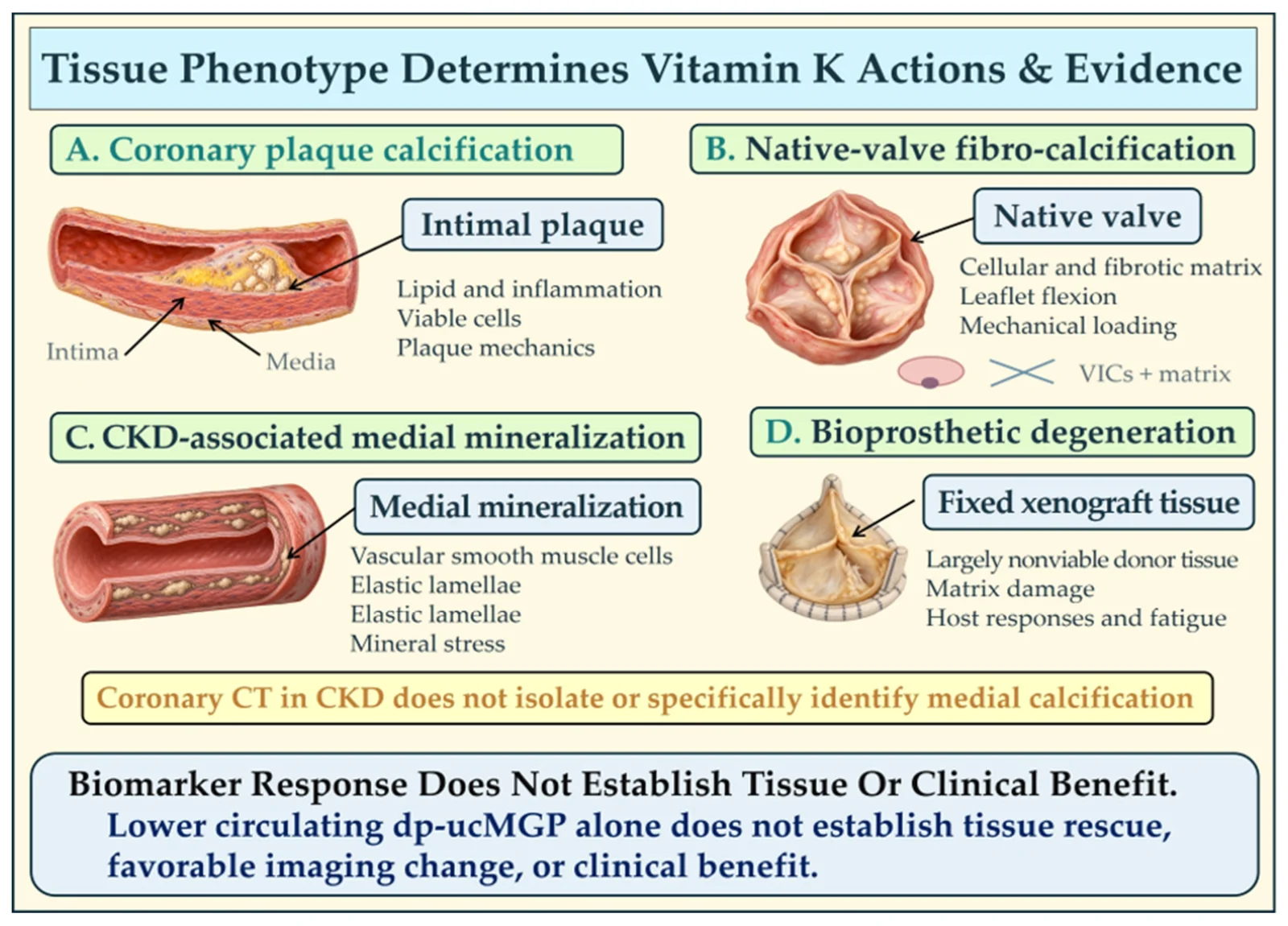
Tissue phenotype influences the interpretation of vitamin K evidence. (**A**) Coronary plaque calcification involves intimal atherosclerotic plaques characterized by lipid accumulation, inflammation, viable cells, and plaque mechanics. (**B**) Native-valve fibro-calcification involves valvular interstitial cells (VICs), extracellular matrix remodeling, leaflet flexion, and mechanical loading. In panel B, the pink oval represents a VIC, while the blue crossed lines represent extracellular matrix fibers. (**C**) Chronic kidney disease (CKD)-associated medial mineralization involves vascular smooth muscle cells, elastic lamellae, and mineral stress. (**D**) Bioprosthetic degeneration involves fixed xenograft tissue, matrix damage, host responses, and mechanical fatigue. These conditions differ in viable cell populations, mechanical loading, and initiating factors. Coronary CT in patients with CKD does not specifically isolate medial calcification. A reduction in circulating dephosphorylated-uncarboxylated matrix Gla protein (dp-ucMGP) alone does not establish tissue-level rescue, favorable imaging changes, or clinical benefit. The original explanatory schematic integrates mechanistic distinctions with representative trials; it is not a pooled comparison of treatment effects [36,59].

Cardiovascular tissues are especially susceptible to vitamin K insufficiency. When vitamin K limits the endogenous pathways that prevent calcification, MGP accumulates in its inactive, dephosphorylated, uncarboxylated (dp-ucMGP) form, allowing mineralization [3,13]. As a result, dp-ucMGP is a recognized marker of low vitamin K status and increased vascular calcification risk [3,53,56]. Two findings support this mechanism. First, warfarin, a pharmacologic VKORC1 inhibitor, disrupts the vitamin K regeneration cycle and raises the risk of arterial and valvular calcification [12,57]. Second, rat models with a *VKORC1* missense mutation and limited vitamin K intake developed extensive medial arterial calcification in multiple vascular beds [39]. Vitamin K supplementation prevented these effects [13,39]. In the Framingham cohorts, higher levels of inactive MGP were also linked to arterial stiffness and incident heart failure with preserved ejection fraction [58]. These findings provide a biologic rationale for testing whether menaquinone supplementation can restore protection against calcification. Figure 3 illustrates vitamin K supplementation across cardiovascular calcification phenotypes.

## 4. Mechanistic Rationale for Vitamin K_2_ in Cardiovascular Calcification

Section 3 outlined the biological mechanisms by which vitamin K_2_ supports the γ-carboxylation and functional activation of matrix Gla protein (MGP). The following section examines clinical and experimental evidence linking vitamin K status to cardiovascular calcification, distinguishing established findings from those that remain inferential. Additionally, the potential biological synergy between vitamins D and K is discussed, with particular attention to their complementary roles in calcium metabolism and the regulation of vascular calcification.

### 4.1. Matrix Gla Protein Activation

The biology of matrix Gla protein (MGP) was discussed in previous sections. We now examine whether supplemental menaquinone improves circulating biochemical markers of vitamin K-dependent MGP carboxylation in humans. The available evidence demonstrates consistent, dose-dependent outcomes. In a randomized, double-blind trial involving healthy adults, twelve weeks of MK-7 supplementation reduced circulating dephosphorylated-uncarboxylated MGP (dp-ucMGP) by 31% at 180 µg/day and by 46% at 360 µg/day, whereas the placebo group showed no change [60]. In patients on hemodialysis, Westenfeld et al. (45–360 µg/day for 6 weeks) and Caluwé et al. (360–1080 µg thrice weekly for 8 weeks) both demonstrated graded dp-ucMGP reductions, and a separate single-arm hemodialysis cohort confirmed a significant fall with 360 µg/day over 4 weeks [61,62,63]. These findings indicate improved biochemical vitamin K status, but circulating measurements do not directly quantify active MGP within cardiovascular lesions.

Despite a robust biomarker response, the imaging findings were not consistent with the biomarker findings [64]. In a randomized ^18^F-sodium-fluoride PET trial in patients with diabetes, six months of MK-7 lowered dp-ucMGP as expected, yet femoral arterial calcification activity trended upward (*p* = 0.06) [64].

### 4.2. Inhibition of Osteogenic Transformation

The osteogenic switch of vascular smooth muscle cells and valvular interstitial cells is often cited as a potential target of vitamin K [22]. However, this is primarily a downstream effect mediated by MGP and its inhibitory effect on Bone Morphogenetic Protein-2, the main driver of osteogenic trans-differentiation [3,11]. BMP-2 downregulates contractile markers and upregulates RUNX2, msh homeobox 2, and β-catenin to drive mineralization, a process inhibited by vitamin K-dependent MGP [22,26].

Additional vitamin K-dependent mechanisms beyond MGP have been proposed, but the evidence remains predominantly preclinical. In cultured rat VSMCs, MK-4 preserved the contractile phenotype and inhibited osteogenic trans-differentiation through a γ-glutamyl-carboxylase-dependent mechanism [65]. In a separate rat VSMC model, vitamin K_2_ reduced calcification and VSMC apoptosis through the growth arrest-specific protein 6/Axl receptor tyrosine kinase/protein kinase B pathway [66]. These hypotheses require in vivo confirmation, and until then the evidence indicates that the anti-osteogenic effect of vitamin K_2_ is chiefly MGP-dependent.

### 4.3. Anti-Inflammatory and Antioxidant Effects

Chronic inflammation is a trigger for osteogenic transformation [1,22]. Vitamin K has proposed anti-inflammatory and antioxidant effects. It has been reported to downregulate NF-κB signaling and pro-inflammatory cytokine production in cells [67]. However, the clinical anti-inflammatory benefit of menaquinone supplementation remains uncertain. Any such effects may be difficult to separate from the downstream consequence of reduced calcium burden [63].

A separate noncanonical mechanism involves ferroptosis suppressor protein 1-dependent reduction in vitamin K to a radical-trapping hydroquinone that suppresses ferroptosis in experimental systems [68]. This does not establish a clinically effective anti-inflammatory or anti-calcification dose of MK-7.

### 4.4. Crosstalk with Vitamin D and Calcium Metabolism

Vitamin D enhances intestinal calcium absorption and may increase matrix Gla protein (MGP) synthesis; however, MGP activation requires sufficient vitamin K status. Consequently, vitamin D supplementation may be inappropriate when vitamin K is insufficient [3,69]. Observational studies recommend caution with high-dose vitamin D supplementation in individuals with vitamin K deficiency, as this may increase the risk of vascular calcification [69]. Furthermore, elevated concentrations of active vitamin D_3_ (intracellular calcitriol) can promote vascular smooth muscle cell (VSMC) osteogenic differentiation via a vitamin D receptor-RUNX2 interaction [69,70]. Despite these concerns, there is a strong biological rationale for balanced vitamin D_3_-K_2_ co-supplementation to prevent vascular calcification.

A meta-analysis of eight RCTs (971 subjects) found that vitamin D plus K improved bone mineral density and lowered undercarboxylated osteocalcin more than control regimens [71]. Evidence for the prevention of cardiovascular disease remains limited; one randomized trial of vitamin D-K_2_ co-supplementation showed discordant effects with possible coronary benefit but no effect on valvular disease [15,16].

### 4.5. Biochemical Target Engagement and Tissue Response

Matrix Gla protein (MGP) functions as part of an inhibitor network rather than acting as the sole regulator of mineralization. Elevated circulating dp-ucMGP does not necessarily reflect local vitamin K delivery, adequate MGP abundance, or its independently regulated phosphorylation. Extracellular vesicle (EV)-mediated nucleation, calciprotein particle (CPP) exposure, and ENPP1–PPi/TNAP imbalance may persist even when carboxylation is improved [30,32,33,34,35]. Coronary plaque, native valves, arterial media, and fixed bioprosthetic tissue exhibit differences in viable cell populations, matrix composition, and mechanical loading. These differences provide testable hypotheses for the observed discordance. Consequently, biochemical response, tissue inhibition, imaging changes, and clinical outcomes should be assessed independently and with caution (Figure 3).

## 5. Pre-Clinical Evidence

*In vitro* work provides a mechanistic basis for further testing. Cultured vascular smooth muscle cells (VSMCs) are an important source of MGP in the arterial wall, with three times higher vitamin K epoxide reductase activity than that measured in hepatocytes [3,13]. Elevated extracellular calcium upregulates MGP production. However, poorly carboxylated MGP accumulates in calcified lesions, consistent with impaired MGP activation during calcification [13,22]. Adding vitamin K to VSMC cultures facilitates MGP γ-carboxylation, which restrains BMP-2-mediated osteogenic transformation [3,22]. Vitamin K has also been reported to antagonize NF-κB signaling in cell systems, the proposed but still unproven anti-inflammatory action discussed in Section 4.3 [3].

### 5.1. Cell Culture Studies

In human aortic valve interstitial cells (HAVICs) isolated from patients with aortic stenosis, *MGP* knockdown induced spontaneous calcification during prolonged culture, supporting a protective role for MGP [45]. In a separate HAVIC model, warfarin promoted calcification in high-phosphate medium. Notably, MK-4 also accelerated calcification, both independently and in combination with warfarin, via signaling pathways involving pregnane X receptor (PXR), BMP-2, and alkaline phosphatase [72]. These in vitro findings indicate that the effects of specific vitamin K forms are influenced by cell type and experimental conditions, and they caution against generalizing the effects of MK-4 across vascular and valvular tissues. The context-dependent PXR effect observed does not provide evidence of clinical harm from MK-7 or nutritional vitamin K_2_ supplementation. The Yang et al. study should be considered alongside its published correction regarding an image in Figure 2A [72].

### 5.2. Animal Models

Most animal evidence is primarily derived from the warfarin model. Warfarin inhibits VKORC1, thereby disrupting the vitamin K cycle and resulting in time- and dose-dependent calcification of the elastic lamellae in rat arteries [57]. The effects of vitamin K_1_ and K_2_ supplementation differ; vitamin K_2_ (MK-4) achieves approximately threefold higher local concentrations in aortic tissue compared to vitamin K_1_ and inhibits vascular calcification [73]. In this model, high dietary supplementation with vitamin K_1_ or MK-4 leads to partial regression of calcification, evidenced by a 50% reduction in arterial calcium content and restoration of vessel distensibility towards control values [47]. Similarly, high-dose MK-7 has been shown to inhibit cardiovascular calcification and to increase aortic *MGP* mRNA expression by approximately tenfold (*p* < 0.05) [74].

Experimental studies demonstrate that CKD establishes a strongly procalcific environment. In rat models with CKD, therapeutic warfarin levels result in accelerated vascular calcification compared to healthy controls. In contrast, high dietary vitamin K_1_ intake reduces calcification [41]. In rats with adenine-induced CKD, elevated dietary vitamin K_1_ increases tissue vitamin K concentrations and mitigates the progression of vascular calcification [41]. Combined with genetic evidence that *MGP*-knockout mice die within two months due to arterial calcification causing blood vessel rupture (Section 1.2), these animal models indicate that vitamin K sufficiency inhibits, while deficiency promotes, mineralization [11].

### 5.3. Limitations of Pre-Clinical Models

The animal model studies are mechanistically persuasive, but their translation to human therapeutics has barriers. First, animal models are rapid and often aggressive- adenine-driven uremia and warfarin-induced calcification led to rapid vascular medial elastocalcinosis (Mönckeberg-type); whereas humans have slowly progressive intimal calcification in atherosclerotic coronary vessels and slowly accreting calcium in heart valves [22,57]. A compound that regresses weeks-old medial elasto-calcinosis in a warfarin-treated rat may not act on decades-old intimal plaque in humans [23,47].

Second, the dosages administered in rat models cannot be directly extrapolated to humans. Animal studies utilized supraphysiologic doses of vitamin K supplementation, approximately 100 µg/g of diet, which greatly exceed typical human supplemental exposure [41,47]. Third, the disease process simulated in animal models spans only weeks, whereas the subclinical disease process in humans develops over decades. Endpoints in animal models included arterial calcium content, MGP carboxylation status, and aortic *MGP* mRNA, rather than clinical events. As Section 4 emphasizes, these surrogate endpoints may not correspond to imaging or clinical outcomes.

Finally, species-specific metabolism of vitamin K (e.g., vitamin K_1_ to MK-4) can result in different tissue partitioning and outcomes. The paradoxical effect of MK-4 in the HAVIC-PXR model illustrates the limitations of extrapolation across tissue types and vitamin K_2_ forms [22,41,47,57,73].

## 6. Observational and Human Evidence

Circulating dp-ucMGP serves as an indicator of functional vitamin K status; however, it does not directly reflect local MGP synthesis or activity within cardiovascular tissues. Dephosphorylated-uncarboxylated MGP (dp-ucMGP) levels act as a surrogate marker for vitamin K status and have been linked to adverse clinical outcomes in prospective studies. In the population-based Bruneck Study, elevated dp-ucMGP was associated with increased incidence of cardiovascular disease and all-cause mortality over a 15.5-year follow-up period. Notably, each standard-deviation increase in dp-ucMGP corresponded to a 40% higher mortality risk after multivariable adjustment, even when dietary quality was considered [43].

### 6.1. Biomarkers of Vitamin K Status

In a prospective cohort of 799 patients with chronic stable vascular disease, those in the highest dp-ucMGP quartile had approximately 89% higher all-cause mortality and 88% higher cardiovascular mortality over a median follow-up of 5.6 years [75]. In a separate Flemish cohort study, higher dp-ucMGP was associated with a lower incidence of coronary events [76]. Mendelian randomization analysis supported a possible causal association for noncancer mortality and an inverse relation with coronary events but not for total or cardiovascular mortality, complicating the interpretation of dp-ucMGP as a universal cardiovascular risk marker [76]. In MESA, higher inactive MGP was associated with cardiovascular disease and mortality, though the effect was concentrated in a younger subgroup aged 45–53 years and not in older participants [77].

Several limitations are present in these studies. Dp-ucMGP serves as a status marker rather than a direct indicator of tissue calcification, and assay thresholds and reference ranges lack full standardization across demographic groups. Although observational associations may be robust, they cannot replace evidence from interventional studies. Table 2 provides a comparison of evidence from animal models and human clinical studies.

### 6.2. Coronary Calcification Studies

The Rotterdam Study found favorable associations for menaquinone (vitamin K_2_), but not for phylloquinone (vitamin K_1_). Among 4807 participants, higher dietary menaquinone intake (>32.7 µg/day; median 40.9 µg/day in the highest tertile) was linked to significantly lower coronary heart disease mortality (relative risk approximately 0.43 for the highest versus lowest tertile), reduced all-cause mortality, and about half the odds of severe abdominal aortic calcification. In contrast, vitamin K_1_ intake was not related to any outcome [78]. More recent prospective data from Denmark associated higher dietary intakes of both phylloquinone and menaquinones with lower rates of atherosclerotic cardiovascular disease hospitalization, although the possibility of residual confounding cannot be excluded [79].

The UK Biobank analysis presents a contrasting dietary signal. Among 112,111 participants initially free of ASCVD and aortic stenosis, K_1_ intake was associated with lower ASCVD incidence in the reported comparison, while higher K_2_ intake was linked to a modestly higher incidence [80]. Differences in food sources, dietary measurement, and residual confounding restrict causal interpretation. These findings do not indicate that K_2_ supplements cause harm, and ASCVD incidence does not directly measure CAC progression.

Randomized interventional coronary evidence has expanded recently, although the trials remain heterogeneous in population, baseline CAC burden, vitamin K formulation and dose, vitamin D co-supplementation, and statistical power. Previous RCTs were largely null or showed effects in smaller subgroups [81,82]. These trials varied in population, duration, vitamin K formulation and dose, vitamin D co-supplementation, and statistical power [83]. The AVADEC CAC sub-study (MK-7 720 µg + vitamin D) showed no effect on CAC in the total group [16] and Kurnatowska et al. found only a non-significant trend confined to patients with lower baseline calcification [82].

A trial of vitamin K_1_ [81] similarly slowed CAC progression only in an adherent subgroup [81]. VitaK-CAC is the first dedicated CAC-MK-7 trial to demonstrate a modest attenuation of CAC score and calcium mass progression over 24 months of MK-7 360 µg supplementation versus placebo [14]. CAC nevertheless increased during follow-up, and the trial did not establish plaque regression or clinical benefit. The trial randomized 180 patients, of whom 167 were included in the reported analysis (85 MK-7 and 82 placebo), 78% of whom were receiving statins; adherence was approximately 80%, and the study was not designed to assess clinical cardiovascular outcomes. The ongoing Inter-Vitamin K trial will further evaluate the effect of MK-7 on CAC progression in a larger population [83].

DANCODE randomized 400 participants with CAC ≥ 400 Agatston units; 398 were included in the reported analysis. MK-7 720 µg/day plus vitamin D_3_ 25 µg/day for 24 months produced less mean CAC progression than placebo (196 versus 248 units; between-group difference −52, 95% CI −79 to −24) [59]. The result extends the coronary imaging signal to substantial baseline calcification, but does not establish plaque regression, an independent vitamin D effect, or fewer clinical events.

The VitaK-CAC editorial by Blaha and Choi and a related commentary by Liu and colleagues place the positive imaging signal as promising, but not definitive, since imaging is a surrogate endpoint used as a substitute for patient-centered clinical outcomes [84,85]. These interpretations are not additional trials or independent efficacy estimates. Neither supports replacing statins, nor does slower calcium accumulation necessarily improve outcomes.

### 6.3. Valvular Disease Studies

A small, open-label, randomized proof-of-concept trial reported slower progression of aortic valve calcification with vitamin K_1_ over 12 months; however, the limited sample size, open-label design, and short follow-up period restrict definitive interpretation [86]. Larger randomized studies in patients with established aortic valve calcification have not demonstrated significant benefit regarding calcification progression or valve hemodynamics (AVADEC; MK-7 720 µg/day plus cholecalciferol 25 µg/day, for 24 months). Together, these findings do not establish that vitamin K slows aortic valve calcification.

While some coronary studies and subgroup analyses have indicated potential benefits, the coronary findings remain inconsistent [15,16]. Table 3 summarizes the principal human trials of vitamin K supplementation for cardiovascular calcification. These studies exhibit several limitations. Most trials included participants with pre-existing valvular calcification, as exemplified by AVADEC, which required AVC ≥ 300 AU [15]. Additionally, AVADEC enrolled only men aged 65 to 74 years. Recent spatial and single-cell analyses in humans have identified sex-associated VIC and macrophage programs, highlighting the limitation of extrapolating these results to women [36]. However, these tissue findings do not demonstrate a sex-specific treatment effect.

As discussed in Section 2, calcification constitutes a self-perpetuating cycle that, once established, may respond less effectively to vitamin K supplementation. These findings support the hypothesis that earlier stages of disease are more amenable to intervention than established calcification, emphasizing the need for prospective studies (Section 2). An untested hypothesis proposes that vitamin K_2_ may be more effective in early aortic sclerosis or among high-risk populations, such as patients with CKD, individuals with biochemical vitamin K insufficiency, or long-term users of vitamin K antagonists, rather than as a treatment for established aortic stenosis (Table 3).

### 6.4. Vitamin K_2_ and Prosthetic Heart Valves

The biological basis for vitamin K_2_ supplementation is the γ-carboxylation-mediated activation of MGP, which is synthesized by living valvular interstitial cells [1,40]. Hence, the relevance of vitamin K_2_ in prosthetic valves depends on the prosthesis type- mechanical or bioprosthetic.

Mechanical valves do not experience the durability-limiting leaflet mineralization observed in bioprosthetic valves. The primary concern with mechanical valves is the interaction between vitamin K_2_ and warfarin, a vitamin K antagonist [90]. Patients with mechanical valves require long-term vitamin K antagonist (VKA) therapy to prevent valve thrombosis and systemic embolism. Vitamin K supplementation can modify VKA requirements or decrease the international normalized ratio (INR); therefore, supplementation should be accompanied by regular INR monitoring and warfarin dose adjustments [90,91,92]. Small clinical trials have indicated that low-dose vitamin K may reduce INR variability in selected warfarin users. However, this effect may result from closer INR monitoring and improved adherence in these patients. Additionally, these trials did not assess prosthetic valve calcification or durability [91,92].

Surgically implanted and transcatheter bioprosthetic valves undergo degeneration through leaflet tearing, fibrosis, and calcification [4,42,93,94,95,96]. The mechanisms underlying fibrocalcific changes in bovine and porcine tissues partially overlap with those of native valve calcification, as glutaraldehyde-fixed xenograft tissue is predominantly nonviable and serves as a substrate for passive hydroxyapatite mineral nucleation [4]. Explanted bioprosthetic tissue demonstrates the presence of vitamin K-dependent γ-carboxyglutamate-containing proteins, osteocalcin, osteopontin, and other matrix proteins associated with mineralization [40,46,97]. These observations support additional mechanistic plausibility of supplemented vitamin K_2_, although they do not confirm its efficacy in preventing structural valve degeneration [98].

The evidence regarding vitamin K_2_ and vitamin K antagonists (VKAs) in recipients of bioprosthetic valves remains inconclusive. Early studies indicated reduced calcification in porcine bioprostheses among patients treated with warfarin. However, an experimental study using a bovine shunt model demonstrated that warfarin did not prevent mineralization or affect the accumulation of γ-carboxyglutamate-containing proteins [46,99]. Conversely, a recent observational cohort study involving 1387 patients who underwent surgical bioprosthetic aortic valve replacement identified warfarin use as an independent risk factor associated with hemodynamic valve deterioration after five years [44]. The heterogeneity in study design, valve technology, and indications for anticoagulation complicates interpretation. To date, no study has established a causal relationship between vitamin K-dependent inhibition of calcification and the durability of prosthetic valves.

Furthermore, evidence from RCTs in native valves cannot be directly applied to bioprosthetic valves. Currently, no trials have evaluated vitamin K_2_ for the prevention of calcification or structural degeneration in surgical or transcatheter bioprosthetic valves. Initial studies should assess the safety and biological effects of vitamin K_2_ on bioprosthetic valve calcification and structural deterioration. Should these studies demonstrate benefit, larger trials designed to assess clinical outcomes would be justified.

### 6.5. Confounders, Potential Reverse Causation, and Solutions

The observational studies, though biologically coherent, must be interpreted with caution due to the reasons mentioned below [76,77,78]. Menaquinone-rich foods, such as organ meats, fermented foods, and natto, are more commonly consumed by individuals from higher socioeconomic backgrounds who generally maintain better overall health. Therefore, the observed benefits may be attributable not solely to vitamin K intake but also to a micronutrient-rich diet. Low vitamin K status and extensive cardiovascular calcification share risk factors, including advanced age, renal dysfunction, frailty, and diabetes, each of which can independently elevate dp-ucMGP levels and promote vascular mineralization.

While CKD leads to significant vitamin K deficiency and accelerates calcification, the relationship between dp-ucMGP and calcification risk may reflect the severity of renal disease rather than a direct effect of vitamin K. Although some studies, such as the Bruneck Study, adjust for overall dietary quality to address this issue, residual confounding cannot be excluded in observational research designs [43].

Severe atherosclerotic or valvular disease may increase the demand for local carboxylation, resulting in elevated circulating levels of dp-ucMGP [3,43]. In this context, the biomarker may be elevated due to increased demand rather than disease progression. Cross-sectional and prospective studies are especially vulnerable to this type of reverse causation. Individuals who consume vitamin K-rich foods or take supplements are often more health-conscious, engage in regular physical activity, smoke less, adhere more consistently to medications, and utilize preventive care more frequently. These behaviors independently reduce cardiovascular risk, thereby confounding observational comparisons between supplement users and non-users due to the underlying decision to supplement.

Randomized trials and large, rigorously designed prospective studies are necessary to address these confounders. Observational evidence can support biological plausibility and fulfill Bradford Hill’s criteria for causality [76]. Future trials should document baseline vitamin K status, employ a biologically justified dose, assess adherence and biochemical target engagement, ensure adequate follow-up, and evaluate phenotype-specific imaging and clinical outcomes. 

The optimal assessment of any micronutrient requires large, community-based longitudinal observational clinical studies, ideally conducted in populations known to be deficient in the index nutrients. Entry criteria should include measurement of baseline micronutrient status, levels achieved in the blood (as in the case with 25OHD), administration of an appropriate dose intended to achieve a clinically meaningful effect, assurance of adherence, and extended follow-up to observe clinical outcomes.

## 7. Clinical Interpretation and Unanswered Questions

Vitamin K supplementation consistently improves functional vitamin K status and is reflected in biochemistry, but its effects on imaging vary by calcification phenotype [100]. VitaK-CAC [14] and DANCODE [59] provide positive coronary imaging signals at moderate and severe baseline calcium burdens, respectively. Published pooled analyses also report a small CAC signal [101] but do not establish fewer cardiovascular events. Earlier or less calcified disease is therefore a hypothesis for enrichment, rather than a proven predictor of response. The native-valve evidence and its limitations are summarized in Section 6.3.

### 7.1. Patient Selection and Adjunctive Therapy

Future studies should select participants by calcification phenotype and baseline vitamin K status. The InterVitaminK protocol evaluates MK-7 in participants with CAC ≥ 10 AU [83] efficacy in early aortic sclerosis remains unproven [15]. Vitamin K supplementation remains investigational as an adjunct to established cardiovascular prevention. Differences in MK-4 and MK-7 metabolism and tissue effects preclude assuming equivalent efficacy.

Certain drug interactions with vitamin K1 and K2 and their impact on study outcomes merit attention. First, statins reduce the risk of major adverse cardiac events but paradoxically increase coronary vascular calcification [23]. Statins transform the lipid-rich, low-attenuation plaque into denser, calcified plaque, which stabilizes it. Therefore, statins should not be withheld solely because CAC increases during therapy [2,23]. VitaK-CAC’s cohort had 78% statin use, which was also noted by its authors to possibly influence calcification [14,23].

Second, vitamin K1 supplementation directly affects PT-INR levels in warfarin users and may cause sub-therapeutic levels if not specifically managed [91,92]. Although animal models suggest that vitamin K antagonists accelerate calcification, this has not been confirmed in human studies [12,57]. In the IRIVASC trial, 192 anticoagulated patients randomized to rivaroxaban or phenprocoumon showed no difference in coronary or valvular calcification progression at 24 months across Agatston, volume, and mass scores, despite the expected divergence in dp-ucMGP [102]. The authors concluded that their data do not support VKA-mediated acceleration of calcification [102]. Similarly, the Valkyrie study found that discontinuing VKA did not slow calcification [87]. Whether direct oral anticoagulants offer a ‘calcium-sparing’ benefit remains unproven in interventional studies.

In contrast to randomized evidence, the 2026 Danish serial-CT study found that long-term VKA exposure, but not direct oral anticoagulant exposure, was associated with greater CAC progression [103]. However, confounding by indication remains possible, and this association alone does not justify changing anticoagulation solely to alter calcium scores. Although prospective observational studies and several randomized trials suggest that vitamin K_2_ is effective in lowering CAC risks, menaquinone is not currently recommended in any clinical guidelines for improving “arterial” or “cardiovascular” health.

### 7.2. High-Risk Populations

Several populations combine vitamin K_2_ insufficiency and high calcification burden, making them appropriate targets for vitamin K_2_ supplementation (Table 4).

***Chronic kidney disease:*** a combination of high calcification burden, vitamin K_2_ deficiency, and elevated dp-ucMGP several-fold; menaquinone supplementation in this population had little beneficial effect [48,106,107]. The Valkyrie study included 132 hemodialysis patients with atrial fibrillation who were randomized to a vitamin K antagonist, rivaroxaban, or rivaroxaban plus high-dose vitamin K_2_ (2.0 mg, thrice weekly) for 18 months. While the vitamin K_2_ status improved in the K_2_ arm, changes in calcium scores and pulse wave velocity in the coronary artery, thoracic aorta, and cardiac valve did not differ between the three groups [87].

Trevasc-HDK, an RCT (*n* = 178) of maintenance hemodialysis patients randomized to MK-7 360 µg three times weekly or control for 18 months, with CAC as the primary outcome and AVC, carotid-femoral pulse wave velocity, dp-ucMGP and major adverse cardiac events as secondary outcomes, showed that CAC scores did not differ between groups [48]. A plausible explanation is persistent failure of mineral buffering: uremic CPPs can mature toward crystalline secondary particles, while depleted fetuin-A and GRP and procalcific EV activity promote VSMC mineralization [31,32,33]. This mechanism, described in Section 2.3, could persist despite improved MGP carboxylation; it has not been established as the cause of the null trial results. Nevertheless, in a rat model of renal failure, high vitamin K_2_ combined with phosphate binders significantly attenuated vascular calcification in the thoracic and abdominal aorta [108].

Additional dialysis evidence includes iPACK-HD, a feasibility-focused oral K_1_ trial with improved vitamin K status but no demonstrated CAC benefit [88], and the 2025 intravenous K_1_ trial, in which the reported progression risk ratio was 0.45 (95% CI 0.18–1.15), leaving substantial uncertainty [89]. A dialysis-specific meta-analysis of 11 trials and 830 participants found no significant pooled calcification or mortality benefit and predates the 2025 trial [109]. These findings do not establish superiority of one vitamin K form or route.

In diabetes mellitus, the same dissociation was shown, where six months of MK-7 lowered dp-ucMGP but did not reduce femoral arterial calcification activity on fluorine-18 sodium fluoride PET (Section 4.1). Older adults have declined vitamin K status and high calcification burden (mechanism detailed in Section 2.3), but this group remains largely untested.

Long-term vitamin K antagonist users: Valkyrie study extension phase showed that cardiovascular event rates were substantially lower in both rivaroxaban arms versus VKA (hazard ratios ~0.41 and ~0.34). Nevertheless, the rivaroxaban-plus-K_2_ arm gained little over rivaroxaban alone, indicating that though benefit was derived from withdrawing the vitamin K antagonist, the study was not powered to isolate the effect of MK-7 on cardiovascular outcomes [104].

Early aortic valve calcification: well-powered, interventional studies including a cohort with early aortic sclerosis, the prime target for menaquinone supplementation, are required to assess hard clinical endpoints in this population [15].

### 7.3. What Remains Uncertain

While animal studies are convincing, in human trials, causality remains uncertain [12]. Although the Flemish Mendelian-randomization study established a causal relation between high dp-ucMGP levels and selected adverse clinical outcomes, it does not establish whether the biomarker is necessarily modifiable [76]. Mendelian-randomization findings do not establish that vitamin K supplementation will modify cardiovascular outcomes. MK-7 supplementation consistently improves dp-ucMGP, but its effects on imaging endpoints have been inconsistent across phenotypes and trials [15,48]. Vitamin K deficiency or antagonism may accelerate calcification, but restoring vitamin K levels may not halt or regress the process once established. The following aspects of vitamin K_2_ supplementation for cardiovascular calcification remain largely unanswered:

***Optimal form***: the MK-7 form of vitamin K_2_ has the longest half-life and high extrahepatic delivery; hence, it is commonly used in clinical trials. While animal models showed regression of calcification with MK-4 supplementation, *in vitro* studies with human valve interstitial cells showed accelerated calcification through PXR agonism [72]. Whether these vitamin K_2_ subtypes are interchangeable, complementary, or antagonistic is to be determined in head-to-head trials.

***Optimal dose***: A dose–response relationship is established for the biomarker (e.g., 31% dp-ucMGP reduction at 180 µg/day versus 46% at 360 µg), but not for any human clinical trials or imaging endpoints. Trials have used 90 to 2000 µg with different rationales for dose selection. In dialysis, even 2.0 mg thrice weekly has been used, which failed to normalize dp-ucMGP. This raises questions about adequate dosing and its feasibility in populations with the highest need.

On the other hand, some scientists believe that in patients with established cardiovascular calcification, it is necessary to use pharmacological doses of menaquinone supplementation. In an open-label study, adults with CAC were given MK-4 (45 mg/day) for one year. While coronary calcium increased by 14%, arterial stiffness was reduced in those with biochemical vitamin K deficiency [110]. Pharmacological vitamin K regimens have been investigated in patients with established cardiovascular calcification, including higher doses like menaquinone-4 at 45 mg/day with varied protocols, but these studies failed to have reliable recruitment criteria and statistical power. Evidence is currently insufficient to establish an optimal dose or to conclude that doses above 20 mg/day are necessary [110,111].

***Vitamin D_3_-K_2_ co-supplementation***: currently, there are no guidelines for a specific beneficial vitamin D_3_-K_2_ ratio. The AVADEC trial tested the combination of 720 µg MK-7 plus 25 µg vitamin D, which was null for cardiovascular outcomes [15]. Whether a different vitamin D_3_ dose, vitamin D status, or magnesium status modifies the response to vitamin K_2_ has not been established [15].

***Duration of supplementation***: clinical trials in this domain have run for 6 to 36 months, whereas the disease process spans over decades. The VitaK-CAC findings suggest that 24 months may be the minimum duration to detect differences in selected calcification imaging endpoints; whether longer treatment is required to influence clinically meaningful outcomes remains unknown [14]. However, whether the effect is sustained or attenuated over time is unknown. RenaKvit’s observation that dp-ucMGP suppression was not maintained over two years raises the possibility of biological escape, though adherence and assay variability should be considered [105] and the dp-ucMGP values remained lower than the placebo throughout the two years.

***Subject selection:*** trial results differ by phenotype; DANCODE also reported an imaging effect with severe baseline coronary calcification [59]. Whether this is applicable across doses, populations, and durations is to be determined.

***Valve vs. coronary artery discordance***: this is proven at three levels- mechanistic (different cellular mediators of MGP production and calcification, hemodynamics), *in vitro* (MK-4 mediated PXR agonism and accelerated valvular calcification), and trial data (VitaK-CAC’s positive coronary results versus null valvular trials).

### 7.4. What Future Trials Should Measure

*Hard cardiovascular outcomes*: Future trials should be adequately powered to assess major adverse cardiovascular events and mortality. Imaging measures are surrogate endpoints and may not correspond with biomarker changes or clinical benefit.

***Aortic-stenosis outcomes*:** Valvular trials should assess aortic-valve areas, transvalvular gradient, peak jet velocity, progression to severe stenosis, and valve replacement. These outcomes may diverge from changes in aortic-valve calcium. AVADEC included hemodynamic measures as secondary endpoints in patients with established disease. Future trials should evaluate them as primary outcomes in earlier diseases.

***Safety and drug interactions*:** Trials should stratify participants by anticoagulant use. Vitamin K_2_ may interfere with vitamin K antagonists, while it remains uncertain whether these drugs accelerate calcification in humans. Background statin use should also be accounted for when looking at coronary artery calcification in imaging outcomes.

*Biomarker-guided design*: dp-ucMGP may help identify vitamin K insufficiency and monitor biological response. It should not be treated as a surrogate for reduced calcification or improved clinical outcomes. A decrease in dp-ucMGP shows response to vitamin K_2_ supplementation, not clinical efficacy. In CKD, serum calcification propensity, assessed by T50, could inform exploratory enrichment or stratification [31,32,33]. T50 is not a validated vitamin K treatment threshold or a target.

***Dietary and microbiome assessment*:** Studies incorporating gut-microbiome analyses should include detailed assessment of habitual diet and control for dietary confounding [112].

### 7.5. Practical Implications

The effects of vitamin K_2_ are mechanistically plausible, and preclinical and observational evidence is supportive. However, RCTs have failed to demonstrate consistent clinical or imaging benefits. Supplementation improves vitamin K status and lowers dp-ucMGP, but these changes do not reliably translate into clinical benefit. Trials in established aortic-valve calcification have generally been neutral.

Several factors may account for the reported discrepancies. Notably, unlike synthetic pharmaceutical agents, micronutrients such as vitamin D_3_ and K_2_ are not optimally evaluated using RCTs, as demonstrated in recent review articles [113,114]. RCTs are primarily designed for synthetic medications to facilitate regulatory approval. In contrast, micronutrients and natural products exhibit threshold effects; once maximum benefits are achieved, additional supplementation does not yield further improvements [115]. Furthermore, micronutrients function as network nutrients, requiring multiple cofactors to realize their full potential benefits [116]. Their efficacy is more accurately assessed through longer-term, well-designed prospective studies [115]. However, in addition to observational clinical studies, RCTs can be valuable for evaluating K_2_ in specific contexts, such as valvular calcification [59].

Vitamin K_2_ should not replace key lifestyle changes like smoking cessation, blood-pressure control, glycemic management, physical activity, or a healthy diet, as well as statin therapy. Its potential role as adjunctive therapy in patients with early calcification and biochemical vitamin K insufficiency remains investigational. Patients should understand that vitamin K_2_ is a micronutrient with a plausible biological role, not a proven “arterial decalcifier.” Those taking vitamin K antagonists should use vitamin K supplements only with medical supervision and regular anticoagulation monitoring.

## 8. Conclusions

Vitamin K supplementation improves biochemical indices of functional vitamin K status, particularly circulating dp-ucMGP. However, improvements in these biochemical markers have not consistently resulted in reduced cardiovascular calcification. The VitaK-CAC trial demonstrated a modest attenuation of coronary artery calcification (CAC) progression in patients with symptomatic coronary artery disease and moderate baseline CAC, while the DANCODE study reported slower progression in individuals with severe baseline CAC [59]. In contrast, clinical outcomes from trials involving established aortic valve calcification and advanced chronic kidney disease (CKD) have been disappointing. The clinical significance of modifying CAC progression remains uncertain.

Micronutrient evaluation requires large, well-designed prospective observational studies alongside randomized trials with nutrient-specific entry criteria, adequate dosing, assessment of achieved status and adherence, and sufficient exposure duration [115,117]. No randomized trial to date has demonstrated that vitamin K supplementation reduces cardiovascular events, valve replacement, or mortality, primarily because of the poor study designs and other limitations described in this manuscript and elsewhere. At present, vitamin K2 should not be “routinely” recommended for the prevention or treatment of cardiovascular calcification outside of clinical trials, including for patients receiving vitamin K antagonists. Future clinical studies (preferably larger, longer-term observational clncial studies) should employ robust designs with sufficient sample sizes and focus on specific patient phenotypes. These studies should enrich for functional vitamin K insufficiency, utilize standardized multimodal imaging, and incorporate long-term clinical follow-up.

## Figures and Tables

**Table 2:** Comparison of evidence from animal models and human clinical studies of the vitamin K–MGP axis and cardiovascular calcification.

Preclinical Evidence	Reference	Human Evidence	Reference
Genetic or pharmacologic disruption of the vitamin K–MGP axis promotes vascular and valvular calcification.	Luo et al., 1997 [3,11,22]; Price et al., 1998 [57]	Observational studies associate higher vitamin K_2_ intake with lower cardiovascular risk, although causality is not established.	Geleijnse et al., 2004 [78]
Vitamin K supplementation can prevent or partially reverse mineralization in animal models. Responses vary by vitamin K form, tissue, and model.	Spronk et al., 2003 [73]; Schurgers et al., 2007 [47]	Randomized trials consistently improve vitamin K status but show neutral or inconsistent effects on native aortic-valve and CKD-associated vascular calcification.	Diederichsen et al., 2022; Haroon et al., 2023 [15,48]
Animal-model prevention and regression findings are summarized above; no directly matched preclinical comparison of the coronary trials is available.	See preceding row [47,73]	Slower coronary artery calcification progression may occur in selected populations, but effects on clinical cardiovascular outcomes remain unestablished.	Vossen et al., 2026; Hasific et al., 2026 [14,59]

**Table 3:** Principal human studies of cardiovascular calcification and vitamin K.

Study	Population/Phenotype	Intervention	Follow-Up	Primary Endpoint	Main Result	Biomarker Response	Key Limitations
Rotterdam Study [78]	Cohort; *n* = 4807; community adults; aortic calcification and CHD	Dietary menaquinone and phylloquinone; no intervention	7.2 years	CHD events, mortality and aortic calcification	Higher menaquinone intake was associated with lower CHD mortality and aortic calcification; no association for phylloquinone	Not assessed	Observational; residual confounding; cannot establish supplementation benefit
MESA [77]	Cohort; *n* = 2663; ages 45–84 without baseline CVD	Baseline dp-ucMGP; no intervention	16–18 years	CVD, CHD and mortality	Higher dp-ucMGP predicted events only in participants aged 45–53 years	Dp-ucMGP was the exposure	Observational; single baseline measurement; age-subgroup finding
Shea et al. [81]	RCT; *n* = 388 older adults; CAC	Vitamin K_1_ 500 µg/day plus multivitamin vs. multivitamin	3 years	Change in CAC	Neutral overall; less progression in adherent participants with baseline CAC > 10 AU	Improved vitamin K status	Vitamin K_1_ study; benefit limited to subgroup
Kurnatowska et al. [82]	RCT; *n* = 42; CKD stages 3–5; CAC	MK-7 90 µg/day plus vitamin D_3_ 10 µg/day vs. vitamin D_3_	~9 months	Changes in CAC and carotid intima–media thickness	Favorable trends, but no definitive reduction in CAC progression	Dp-ucMGP decreased	Very small, short trial; limited power
AVADEC [15]	Multicenter RCT; *n* = 365 analyzed; men with AVC > 300 AU	MK-7 720 µg/day plus vitamin D_3_ 25 µg/day vs. placebo	2 years	Change in aortic-valve calcium	No effect on AVC progression or valve hemodynamics	Dp-ucMGP decreased	Men only; established AVC; combined intervention
AVADEC coronary analysis [16]	Secondary analysis; *n* = 304 men with AVC and frequent CAC	MK-7 720 µg/day plus vitamin D_3_ 25 µg/day vs. placebo	2 years	Change in CAC	CAC difference was nonsignificant overall; possible benefit with baseline CAC ≥ 400 AU	Dp-ucMGP decreased	Secondary analysis; subgroup finding; men only
Valkyrie [87]	RCT; *n* = 132; hemodialysis with atrial fibrillation	VKA vs. rivaroxaban vs. rivaroxaban plus MK-7 2000 µg three times weekly	18 months	Calcification and arterial stiffness	No significant imaging or stiffness benefit	Lowest dp-ucMGP with rivaroxaban plus MK-7	Open-label; differing anticoagulants; advanced disease
Trevasc-HDK [48]	RCT; *n* = 178; hemodialysis; CAC and AVC	MK-7 360 µg three times weekly vs. standard care	18 months	CAC score	No benefit for calcification, stiffness, major adverse cardiac events or mortality	Dp-ucMGP decreased	Single center; no placebo; incomplete follow-up
VitaK-CAC [14]	Phase 3 RCT; *n* = 180 randomized; 167 analyzed; symptomatic CAD; CAC 50–400 AU	MK-7 360 µg/day vs. placebo	2 years	CAC score and calcium mass	MK-7 modestly reduced CAC and calcium-mass progression	Plasma MK-7 increased; inverse association with dp-ucMGP	Small trial; dropout; imperfect adherence; surrogate outcomes
iPACK-HD [88]	Pilot RCT; *n* = 86; hemodialysis	Oral K_1_ 10 mg three times weekly vs. placebo	12 months	Feasibility; CAC assessed	Feasible; no demonstrated CAC benefit	Functional status improved	Pilot not powered for definitive imaging or event efficacy
Macias-Cervantes et al. [89]	RCT; *n* = 60; hemodialysis	IV K_1_ 10 mg three times weekly vs. placebo	12 months	CAC progression	RR 0.45 (95% CI 0.18–1.15); inconclusive	Not the basis of the efficacy conclusion	Small single-center trial; CI includes no effect
DANCODE [59]	RCT; 400 randomized; 398 analyzed; CAC ≥ 400 AU	MK-7 720 µg/day + D_3_ 25 µg/day vs. placebo	24 months	CAC progression	Mean increase 196 vs. 248 AU; difference −52 (95% CI −79 to −24)	Biomarker response is not a clinical outcome	Combined intervention; imaging endpoint; no proven event benefit

**Table 4:** Potential clinical settings for vitamin K_2_.

Setting	Rationale	Human Evidence	Position
Symptomatic CAD with CAC 50 to 400 AU	Moderate calcification may remain modifiable.	One RCT found modestly slower CAC progression [14]	Investigational.
Early/subclinical CAC	Earlier treatment may precede self-sustaining mineralization.	Indirect support; InterVitaminK is testing this [83]	Investigational.
Established aortic valve calcification	MGP is expressed in valve tissue.	AVADEC found no imaging or hemodynamic benefit [15]	Not supported.
Bicuspid aortic valve	Earlier disease and distinct biology.	No confirmed definitive efficacy results.	Insufficient evidence.
Nondialysis CKD	Vitamin K insufficiency and calcification are common.	Small trials are neutral or show subgroup signals [82]	Investigational.
Hemodialysis	High dp-ucMGP and calcification burden.	Biomarkers improve; imaging and outcomes generally do not [48,104,105]	Routine use is unsupported.
Diabetes	High calcification risk and functional K insufficiency.	Lower dp-ucMGP without reduced PET calcification activity [64]	Insufficient evidence.
Long-term VKA therapy	VKAs impair MGP carboxylation.	Human calcification evidence is inconsistent [102]	Requires medical supervision.
General prevention	Dietary associations are plausible.	No RCT has shown fewer cardiovascular events.	Not recommended.

***Abbreviations:*** AU, Agatston units; CAC, coronary artery calcification; CAD, coronary artery disease; CKD, chronic kidney disease; MGP, matrix Gla protein; PET, positron emission tomography; RCT, randomized controlled trial; VKA, vitamin K antagonist.

## Data Availability

No new data were created or analyzed in this study. Data sharing is not applicable.

## Abbreviations

The following abbreviations are used in this manuscript:

**Abbreviation:**	**Definition:**
AU	Agatston units
AVADEC	Aortic Valve DECalcification trial
AVC	Aortic valve calcification
BMP-2	Bone morphogenetic protein 2
CAC	Coronary artery calcification
CKD	Chronic kidney disease
CPP	Calciprotein particle
CT	Computed tomography
CVD	Cardiovascular disease
dp-ucMGP	Dephosphorylated-uncarboxylated matrix Gla protein
ENPP1	Ectonucleotide pyrophosphatase/phosphodiesterase 1
EV	Extracellular vesicle
GGCX	Gamma-glutamyl carboxylase
Gla	Gamma-carboxyglutamic acid
GRP	Gla-rich protein
HAVIC	Human aortic valve interstitial cell
INR	International normalized ratio
IRIVASC	Influence of rivaroxaban compared with vitamin K antagonist treatment upon development of cardiovascular calcification
K_1_	Vitamin K_1_ (phylloquinone)
K_2_	Vitamin K_2_ (menaquinone)
MESA	Multi-Ethnic Study of Atherosclerosis
MGP	Matrix Gla protein
MK-4, MK-7, MK-8, MK-9	Menaquinone-4, menaquinone-7, menaquinone-8, menaquinone-9
MK-n	Menaquinone with n isoprenoid units
NF-κB	Nuclear factor kappa B
PET	Positron emission tomography
PPi	Inorganic pyrophosphate
PXR	Pregnane X receptor
RCT	Randomized controlled trial
RUNX2	Runt-related transcription factor 2
T50	Serum calciprotein-particle maturation time
TNAP	Tissue-nonspecific alkaline phosphatase
Trevasc-HDK	Treatment with vitamin K_2_ on vascular calcification in hemodialysis patients
VitaK-CAC	Vitamin K–Coronary Artery Calcification trial
VKA	Vitamin K antagonist
VKDP	Vitamin K–dependent protein
VKORC1	Vitamin K epoxide reductase complex subunit 1
VSMC	Vascular smooth muscle cell

## Appendix A. Narrative Review Literature Update

A systematic search strategy was not used for the original narrative review. For this revision, additional targeted searches were conducted on 30 September 2026 using PubMed-indexed records, primary journal articles, and official trial records relevant to reviewer-identified gaps. Search topics included vitamin K/phylloquinone/menaquinone trials; EVs, CPPs, fetuin-A and T50; ENPP1–PPi/TNAP; spatial and sex-associated valve biology; and named studies and commentaries. Title/DOI and correction checks were also performed. Sources were selected based on their relevance to the review and reviewer concerns. No exhaustive screening, duplicate independent selection, numerical screening flow, or new meta-analysis was conducted. Selection bias remains a limitation.
